# Probiotic *Apilactobacillus Kunkeei* EABW06 from Beeswax Microbiota: A Candidate for Functional Dairy Foods

**DOI:** 10.1007/s12602-025-10817-7

**Published:** 2025-11-11

**Authors:** Mariam Hassan, Yasser Essam Elenany, Ashwak Abdel-Moneim Hassan, Noha A. Ahmed

**Affiliations:** 1 Department of Microbiology and Immunology, Faculty of Pharmacy, Cairo University, Kasr El-Aini Street, Cairo, 11562 Egypt; 2 Department of Microbiology and Immunology, Faculty of Pharmacy, Galala University, New Galala City, Suez Egypt; 3 Department of Economic Entomology and Pesticides, Faculty of Agriculture, Cairo University, 12613, Giza, Egypt; 4 Department of Dairy Science, Faculty of Agriculture, Cairo University, 12613, Giza, Egypt

**Keywords:** Food safety, Dairy fermentation, Caco-2 cytotoxicity, Antimicrobial activity, Antifungal, Acid and bile tolerance

## Abstract

*Apilactobacillus kunkeei*, a fructophilic lactic acid bacterium (FLAB) associated with the honeybee microbiota, has recently gained attention as an unconventional probiotic source. This study evaluated the probiotic, safety, and technological properties of *A. kunkeei* EABW06, isolated from beeswax, to assess its potential for human and biotechnological applications. *A. kunkeei* EABW06 demonstrated gastrointestinal resilience, with > 96% survival at pH 3.0 and tolerance to 0.3–0.7% bile salts. It exhibited broad-spectrum antimicrobial activity against *Escherichia coli* O157:H7, *Salmonella typhi*, and *Clostridioides difficile*. Additionally, *A. kunkeei* EABW06 inhibited fungal pathogens including *Aspergillus fumigatus*, *Aspergillus flavus*, and *Candida albicans*. HPLC analysis revealed acetic, lactic, butyric, and propionic acids as dominant metabolites, correlating with its antimicrobial effects. Safety assessments confirmed no hemolysis, non-cytotoxicity to Caco-2 cells, and sensitivity to clinically relevant antibiotics. In milk fermentation trials, *A. kunkeei* EABW06 reduced pH to 4.5 ± 0.1, produced 0.98 ± 0.03% lactic acid (titratable acidity), and exhibited proteolytic activity (0.384 ± 0.002) measured by spectrophotometric quantification. In conclusion, *A. kunkeei* EABW06 is a safe, robust probiotic candidate with potential applications in biopreservation and dairy biotechnology.

## Introduction

The field of probiotics has garnered increasing attention over the past decade due to mounting evidence of their diverse health benefits. These benefits range from immunomodulation and exclusion of pathogens to preserving mucosal integrity, exerting antioxidant activity, neutralizing toxins, and aiding overall digestion [1–3]. Moreover, probiotics were associated with the alleviation of conditions such as depression and anxiety [4, 5]. Historically, probiotics have been predominantly isolated from fermented and dairy products, as these have long been associated with health-promoting properties [6]. However, the discovery of the gut microbiota and its critical role in host health has expanded the scope of probiotic research. Since the early 2000 s, advances in sequencing technologies have revealed the complexity of the gut microbiome, linking its homeostasis to a wide array of health outcomes, including protection against *Clostridioides difficile* infections, metabolic disorders such as diabetes and obesity, and even mental health conditions [7–9].

Traditionally, probiotic isolation and cultivation have relied on conventional methods that capture only a narrow diversity of strains. Consequently, most characterized probiotics belong to a limited set of genera—particularly lactobacilli such as *Lactobacillus acidophilus*, *Lacticaseibacillus rhamnosus*, *Lacticaseibacillus casei*, *Lacticaseibacillus paracasei*, and *Lactiplantibacillus plantarum*, as well as *Bifidobacterium*, including *Bifidobacterium animalis* and *Bifidobacterium breve* [10–12] —while many former *Lactobacillus* species were reclassified into new genera in the 2020 taxonomic revision [13]. However, the advent of high-throughput sequencing has unveiled a vast array of uncultivable microorganisms that play pivotal roles in maintaining host health, potentially affecting metabolic, gastrointestinal, or mental aspects. This has spurred interest in exploring unconventional sources for novel probiotic strains, including soil, plants, and even insect-associated microbiota [14–16].

Isolating novel probiotic strains often requires innovative cultivation techniques, such as anaerobic culture systems, co-culturing methods, and the use of fortified media, which have been specifically designed to isolate fastidious microorganisms from complex environments like the gastrointestinal tract (GIT) and other niches [17]. These advanced techniques have enabled researchers to overcome the limitations of traditional culturing methods and access previously ‘unculturable’ microbial diversity [18, 19]. Unconventional sources offer the advantage of discovering novel strains and species with unique functional traits, such as enhanced stress tolerance, antimicrobial activity, and niche-specific adaptations [20, 21]. However, the use of such sources also raises concerns regarding the safety and compatibility of these strains for human consumption, necessitating rigorous evaluation of their probiotic potential, including assessments of antibiotic resistance, toxin production, and host interactions [22–24].

One promising yet underexplored source of novel probiotics is honeybee products, which have been used for centuries in traditional medicine for their antimicrobial, anti-inflammatory, and wound-healing properties [25]. Recent studies have identified lactic acid bacteria (LAB) within the honeybee gut microbiota, exhibiting potential probiotic properties [26]. These LAB strains have demonstrated capabilities such as pathogen inhibition and immune modulation, suggesting their suitability as probiotics for human use. Moreover, honey itself has been recognized as a source of beneficial microorganisms, including various LAB and *Bifidobacterium* species, which contribute to its antimicrobial properties [27]. The exploration of honeybee-associated microorganisms as probiotics is relatively nascent, with significant potential yet to be realized. While traditional sources such as dairy products and fermented foods (e.g., yogurt, kimchi, and sauerkraut) have been extensively studied, the unique microbiota of honeybees offers a novel reservoir of probiotic candidates.

Despite the growing interest in honeybee-associated microbiota, certain products such as beeswax and pollen grains remain underexplored as sources of probiotic candidates. In this study, we aimed to isolate and characterize fructophilic lactic acid bacteria (FLAB) from beeswax, pollen grains and beebread, three underutilized niches with potential for novel probiotic discovery. We conducted a comprehensive evaluation of its safety, technological properties, and potential probiotic properties. Our findings contribute to the expanding body of knowledge on unconventional probiotic sources and highlight the potential of FLAB as a promising candidate for the development of innovative dairy products with human health applications.

## Materials and methods

### Isolation, Bacterial Strains, and Growth Conditions

Samples of beebread, pollen grains, and beeswax were collected during the primary clover bloom season (February to April 2024) from eight honeybee colonies (*Apis mellifera carnica* L., Carniolian first hybrid) maintained at the Faculty of Agriculture’s apiary in the Giza Governorate, Egypt. Empty combs were added to the tested colonies to stimulate pollen storage and transform it into beebread. Beebread, pollen grains and beeswax were collected manually from combs using a spatula and forceps and were placed in sterile 2 mL tubes containing phosphate-buffered saline (PBS, pH 7.4) according to Mohammad et al. [28]. For microbial isolation, 1 g of each matrix (beebread, pollen grains, or beeswax) was inoculated, in triplicate, into 30 mL of de Man, Rogosa, and Sharpe (MRS) broth (Oxoid, UK) supplemented with 2% (w/v) fructose using sterile disposable loops. The cultures were incubated in a shaker incubator at 30 °C and 120 rpm for 72 h [29]. After incubation, the cultures were streaked onto MRS agar plates supplemented with 2% (w/v) fructose and incubated at 37 °C for 48 h. Single colonies were selected, isolated, and purified. The purified isolates were preserved in Brain Heart Infusion (BHI) broth (Oxoid, UK) supplemented with 20% (v/v) glycerol and stored at −80 °C for further analysis.

Other strains used in this study were: *Escherichia coli* O157:H7 EDL933, *Salmonella typhi* ATCC 35,664, and a clinical isolate of *Clostridioides difficile* C74A [30]. *C. difficile* C74A was cultivated anaerobically in Reinforced Clostridial Medium (RCM) semi-broth (Oxoid, UK) at 37 °C for 24 h using Anaerogen™ gas packs (Oxoid, UK) in sealed jars. *E. coli* O157:H7 EDL933 and *S. typhi* ATCC 35,664 were propagated aerobically in Mueller-Hinton (MH) broth (Oxoid, UK) under identical temperature and incubation duration. *Aspergillus fumigatus* RCMB 002008, *Aspergillus niger* NRRL 326, *Aspergillus flavus* NRRL 1957 and *Candida albicans* ATCC 25,923 were utilized for antifungal activity. All strains were preserved in Brain Heart Infusion (BHI) broth containing 20% (v/v) glycerol and cryopreserved at −80 °C for long-term storage.

### Identification of Bee beard, Pollen and Bee Wax Isolates

Molecular identification of the isolated strains was performed using partial 16 S rRNA gene sequencing [31, 32]. To prepare overnight cultures, single colonies from freshly streaked MRS agar plates were inoculated into 5 mL MRS broth and incubated in a shaker incubator with 120 rpm at 37 °C for 24 h, these overnight cultures were centrifuged at 13,000 × *g* for 2 min to pellet the cells. The supernatant was discarded, and the cell pellets were treated with 100 µL of lysozyme (10 mg/mL) at 37 °C for 1 h. Total genomic DNA was extracted using the G-spin™ Total DNA Extraction Kit (iNtRON, Korea) according to the manufacturer’s instructions. The 16 S rRNA gene was amplified using universal primers 27 F (5’-AGA GTT TGA TCC TGG CTC AG-3’) and 1492R (5’-CGG TTA CCT TGT TAC GAC TT-3’) [33]. The PCR products were visualized on a 1% agarose gel stained with ethidium bromide. Sequencing was performed by Macrogen Inc. (South Korea), and the resulting sequences were analyzed using BLASTn on the National Center for Biotechnology Information (NCBI) platform against the non-redundant nucleotide (nt) database to identify the isolates.

## Probiotic Properties Assessment

### Antimicrobial Activity

The antimicrobial activity of *Apilactobacillus kunkeei* EABW06, the isolated FLAB selected based on 16 S rRNA identification, was assessed against *E. coli* O157:H7 EDL933, *S. typhi* ATCC 35,664, and *C. difficile* C74A using the agar overlay method as described by Halder et al. [34, 35]. Briefly, overnight cultures of *A. kunkeei* EABW06 were spot-inoculated (≈ 6 mm diameter) onto MRS agar plates and incubated at 37 °C for 48 h. After incubation, the plates were overlaid with soft MH agar (0.8% w/v agar) containing approximately 10⁶ CFU/mL of the respective strain (*E. coli* or *S. typhi*) and incubated at 37 °C for 24 h under aerobic conditions. For *C. difficile*, RCM agar (1.5% w/v agar) was used for the overlay, and the plates were incubated anaerobically at 37 °C for 48 h. The assay was done in triplicate and antimicrobial activity was evaluated by the presence of clear inhibition zones in the overlay layer [30, 36].

### Antifungal Activity

The antifungal activity of *A. kunkeei* EABW06 was assessed using the agar well diffusion method, as described by Tremonte et al. [37]. The tested fungal strains included *Aspergillus fumigatus* RCMB 002008, *Aspergillus niger* NRRL 326, and *Aspergillus flavus* NRRL 1957. Additionally, anti-yeast activity against *Candida albicans* ATCC 25,923 was evaluated. Fungal suspensions (10⁸ CFU/mL) were prepared following the method of Santos et al. [38]. Briefly, fungal colonies were covered with 5 mL of sterile saline solution (0.9%, w/v), and the surface was gently probed with the tip of a Pasteur pipette to release a mixture of conidia and hyphal fragments. The resulting suspension (100 µL) was then spread over the surface of MH agar, and a 6-mm diameter well was aseptically punched into the agar. Then, 100 µL of *A. kunkeei* EABW06 overnight culture in MRS broth was inoculated into the well. The plates were incubated at 30 °C for 48 h. The antifungal activity was determined by measuring the diameter of the inhibition zone in millimeters (mm) using the following formula:

Inhibition Zone (mm) = Diameter of the inhibited growth zone − Diameter of the well.

The experiment was carried out in triplicate, and the antifungal activity was reported as the mean diameter of the zone of inhibition (ZOI) ± standard deviation (SD) [39]. The inhibitory activity of *A. kunkeei* EABW06 against each microorganism was statistically compared to that of the standard antifungal drug using an unpaired t-test.

### Acid Tolerance Test

Acid tolerance was assessed by first adjusting MRS broth to pH 3.0 with 1 N HCl before sterilization. An overnight culture of *A. kunkeei* EABW06 was standardized to an OD₆₀₀ of 0.1 and then inoculated (10% v/v) into 3 mL of acidified MRS broth. Cultures were incubated at 37 °C in a 5% CO₂ incubator (BINDER, Germany). At 0, 1.5, and 3 h, 30 µL aliquots were withdrawn, serially diluted in sterile peptone–saline diluent, and 10 µL of each dilution was spotted onto MRS agar plates. The spotted MRS plates were incubated at 37 °C under 5% CO₂ for 48 h. Survival (%) at each time point was calculated relative to the initial (0 h) colony count. Parallel controls were run in MRS broth adjusted to pH 6.5. The assay was performed in triplicate, and results are reported as mean ± standard deviation (SD) [40, 41].

### Bile Salt Tolerance

To evaluate bile tolerance, MRS broth was supplemented with 0.3% and 0.7% (w/v) bile salts mixture (LobaChemie, India) prior to sterilization. An overnight culture of *A. kunkeei* EABW06 was adjusted to an OD₆₀₀ of 0.1 and inoculated at 10% (v/v) into 3 mL of the bile-supplemented MRS medium. Cultures were incubated at 37 °C in a 5% CO₂ atmosphere, and samples were withdrawn at 0, 1.5, 3, 6, and 24 h for serial dilution in sterile peptone–saline, followed by spotting 10 µL of each dilution onto MRS agar. MRS plates were then incubated at 37 °C under 5% CO₂ for 48 h. Parallel controls were run in MRS broth without added bile salts. The assay was carried out in triplicate, and data are presented as mean ± standard deviation (SD) [41, 42].

## Safety Profiling

### Hemolytic Activity

Overnight culture of *A. kunkeei* EABW06 was spot-inoculated onto blood agar base supplemented with 9% sheep blood and incubated anaerobically at 37 °C for 72 h. Then, the incubated plate was inspected for hemolytic activity, categorized as beta (clear zone), alpha (greenish zone), or gamma (no hemolysis) [43].

### Antibiotic Susceptibility

Antibiotic susceptibility was evaluated using the disc diffusion method on MRS agar [44]. Approximately 10^8^CFU/mL inocula of *A. kunkeei* EABW06 was tested against eight antibiotics: Chloramphenicol (30 µg), Clindamycin (2 µg), Erythromycin (15 µg), Gentamicin (10 µg), Kanamycin (30 µg), Streptomycin (10 µg), Tetracycline (30 µg), and Vancomycin (30 µg). Zones of inhibition were interpreted according to Clinical and Laboratory Standards Institute (CLSI) guidelines [45].

### Cytotoxicity Assay of Strain-Produced Metabolites Using Caco-2 Cell Line

The Caco-2 cell line (VACSERA, Egypt) was cultured in Dulbecco’s Modified Eagle Medium (DMEM; Gibco, USA) supplemented with 10% v/v fetal bovine serum, 100 µg/mL penicillin, and 100 µg/mL streptomycin. Cells were maintained in humidified tissue culture flasks (Greiner, Germany) at 37 °C under 5% CO2 until confluence and detached using 0.25% Trypsin-EDTA (AMRESCO, USA). *A. kunkeei* EABW06 was cultured in MRS broth at 37 °C with shaking (120 rpm) for 30 h. Cell-free supernatant (CFS) was then harvested by centrifugation of the culture at 5200 × *g* for 15 min, followed by sterilization via filtration through a 0.22 μm cellulose acetate membrane filter. The CFS was stored at −20 °C until use.

Cytotoxicity was assessed using the MTT assay [46]. Caco-2 cells were seeded into 96-well plates at 1 × 10⁴ cells/well and cultured overnight. Cells were then exposed to CFS at concentrations of 100%, 50%, 10%, and 5% (v/v), with sterile MRS broth used as a control. After 48 h of incubation at 37 °C under 5% CO₂, dead cells were removed by washing with PBS containing 0.05% Tween 80. Live cells were treated with 25 µL of 0.5% MTT stain per well and incubated for 3–4 h at 37 °C. Intracellular formazan crystals were dissolved using 50 µL of dimethyl sulfoxide (DMSO), and absorbance was measured at 570 nm using an ELISA plate reader (Biotek-8000, USA). Cell survival percentage was calculated as:

Cell survival (%) = (OD of test-treated cells/OD of control-treated cells) ×100.

Results were expressed as mean percent survival ± standard deviation (SD).

## Technological Characteristics

### Milk Fermentation

Fresh skim milk, free of antibiotics and obtained from the herd at Cairo University’s Faculty of Agriculture (Egypt), was sterilized at 115 °C for 10 min. To prepare the inoculum, 0.1 mL of stock culture of *A. kunkeei* EABW06 was transferred into 10 mL of MRS broth supplemented with 2% (w/v) fructose and incubated at 30 °C for 24 h to reach approximately 10⁸ CFU/mL. The activated culture was then inoculated into sterilized skim milk at 1% (v/v) and incubated at 30 °C for 24 h. The resulting fermented milk was used for all subsequent analyses, including pH, titratable acidity, antioxidant activity, proteolytic activity, and organic acid profiling.

### Analysis of Fermentation Profile: pH and Titratable Acidity

The acidification profile of the *A. kunkeei* EABW06 was evaluated by measuring the pH and titratable acidity (TA) of the fermented milk at 0, 4, 8, 12, 16, 20, and 24 h of incubation at 30 °C, following the method described by Abarquero et al. [47]. The pH variation (ΔpH) was calculated as the difference between the initial and final pH values. Titratable acidity was expressed as a percentage of lactic acid, determined according to the AOAC method [48].

### Antioxidant Activity

The antioxidant activity of the fermented milk was assessed using its water-soluble extract (WSE), prepared according to the method of Elzeini et al. [41]. Briefly, 2 mL of fermented milk was mixed with 1 mL of distilled water and 5 mL of 12% trichloroacetic acid (TCA). After standing for 10 min, the mixture was centrifuged at 10,000 × *g* for 30 min. The resulting supernatant (WSE) was collected and used to evaluate in vitro antioxidant capacity by measuring DPPH free radical scavenging activity and ferrous ion (Fe²⁺) chelating ability spectrophotometrically, as described by Li et al. [49]. Antioxidant activity was calculated and expressed as percentages.

### Proteolytic Activity

The proteolytic activity of the fermented milk was determined spectrophotometrically by quantifying free α-amino groups released from casein hydrolysis, using the method of Abushelaibi et al. [50]. The assay is based on the reaction of free amino groups with o-phthaldialdehyde (OPA) in the presence of β-mercaptoethanol, forming a complex with strong absorbance at 340 nm.

### Analysis of Organic Acid Metabolites in Fermented Milk

The organic acid metabolites produced by *A. kunkeei* EABW06 in the fermented milk were characterized using high-performance liquid chromatography (HPLC), following the method described by Parlindungan et al. [51]. A 20 µL aliquot of the fermented milk sample was injected into an Agilent 1200 high-performance liquid chromatography (HPLC) system equipped with a refractive index detector and a REFEX 8 μm 8% H Organic Acid Rezex@ column (Phenomenex, USA). Identification and quantification of organic acids were achieved by comparing the retention times and peak areas of the samples with those of authentic standards run under identical conditions.

### Statistical Analysis

Statistical analyses were conducted using GraphPad Prism software (version 9.0.2; GraphPad Software, Inc., USA). Continuous variables are presented as mean ± standard deviation (SD) derived from three independent replicates. For comparisons between two groups, unpaired Student’s *t*-tests were applied, while one-way or two-way ANOVA followed by Tukey’s post hoc test was used for multi-group comparisons. In all analyses, the threshold for statistical significance was set at *p* < 0.05.

## Results

### Identification of Bacterial Isolates

Four bacterial isolates were identified through partial 16 S rRNA gene sequencing (Table 1). Among these, two isolates (EAPG01 and EAPG02) originated from pollen grains. EAPG01 showed 100% query coverage and identity with *Lacticaseibacillus paracasei* (formerly *Lactobacillus paracasei*) (NCBI accession: SUB15268848). Intriguingly, EAPG02 exhibited 100% identity with both *Lacticaseibacillus paracasei* and *Lacticaseibacillus casei* (SUB15268865), reflecting the high genetic similarity (> 99% 16 S rRNA homology) between these species, which complicates differentiation using 16 S sequencing alone.

**Table 1:** Molecular identification of bacterial isolates using 16 S rRNA gene sequencing

Isolate	Source	Closest Match (Genus/Species)	Query Coverage (%)	Percent Identity (%)	NCBI Submission Number
EAPG01	Pollen grain	*Lacticaseibacillus paracasei*	100	100	SUB15268848
EAPG02	Pollen grain	*Lacticaseibacillus paracasei/casei*	100	100	SUB15268865
EABB05	Beebread	*Lacticaseibacillus paracasei*	96	100	SUB15268868
EABW06	Beeswax	*Apilactobacillus kunkeei*	100	100	SUB15268872

The third isolate, EABB05, isolated from beebread, was identified as *Lacticaseibacillus paracasei* with 96% query coverage and 100% identity (SUB15268868). The fourth isolate, EABW06, derived from beeswax, was unambiguously classified as *Apilactobacillus kunkeei* (100% query coverage and identity; SUB15268872). Based on its taxonomic identification as *A. kunkeei*, a species well established in the literature as a fructophilic lactic acid bacterium, and its relevance to the study’s objectives, only isolate EABW06 was selected for subsequent probiotic, safety, and technological characterization.

## Probiotic Properties Assessment

### Antimicrobial Activity against Gastrointestinal Pathogens

The antimicrobial potential of *A. kunkeei* EABW06 was evaluated against key gastrointestinal pathogens, including *E. coli* O157:H7, *S. typhi* ATCC 35,664, and *C. difficile* C74A, using the agar overlay method. *A. kunkeei* EABW06 demonstrated broad-spectrum antimicrobial activity, evidenced by distinct inhibition zones in the overlay agar against all tested pathogens.

### Antifungal Activity

The antifungal activity of *A. kunkeei* EABW06 against various molds and yeasts is summarized in Table 2. The strain exhibited significant inhibitory effects, with the potency varying by species. The CFS of *A. kunkeei* EABW06 exhibited significantly larger inhibition zones (*p* < 0.05) against *A. fumigatus* RCMB 002008 (23 ± 1.15 mm) and *(A) flavus* NRRL 1957 (29 ± 0.57 mm) compared to the activity of the standard antifungal, Amphotericin (B) In contrast, no inhibitory activity was observed against *A. niger* NRRL 326, a fungus against which the Amphotericin B standard remained effective.

**Table 2:** Antifungal activity of *A. kunkeei* EABW06 against selected molds and yeasts

Tested Microorganism	Zone of Inhibition (mm)± SD	
Molds	*A. kunkeei *EABW06	Standard Antifungal Amphotericin B
*Aspergillus fumigates* RCMB 002008	23 ± 1.15	18.5 ± 0.57*
*Aspergillus flavus* NRRL 1957	29 ± 0.57	15 ± 0.24*
*Aspergillus niger *NRRL 326	0 ± 0	21.43±0.14*
Yeasts		Fluconazole (5 mg/ml)
* Candida albicans *ATCC 10231	18.8± 0.09	22.5± 0.25*

*Statistical significance for the difference between *A. kunkeei* EABW06 and the standard antifungal was determined by an unpaired t-test (*p < 0.05)

Regarding yeast inhibition, *A. kunkeei* EABW06 displayed notable activity against *C. albicans* ATCC 10,231 (18.8 ± 0.09 mm), although this was significantly lower (*p* < 0.05) than the inhibition observed with fluconazole (22.5 ± 0.25 mm). These findings highlight the selective antifungal activity of *A. kunkeei* EABW06, particularly its strong inhibition of *A. fumigatus* and *A. flavus*.

### Resistance to Acidic pH and Bile Salts

**Fig. 1:**
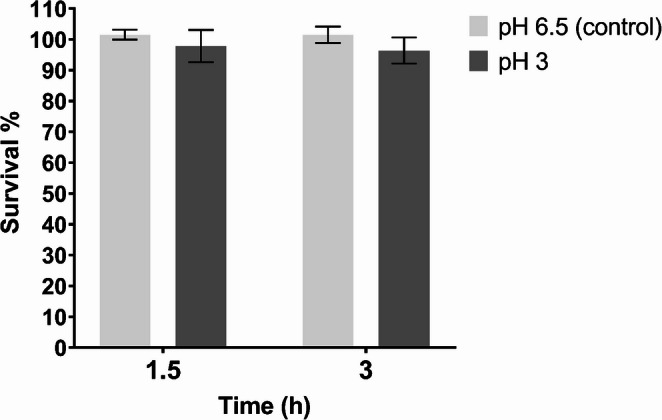
Acid tolerance of *A. kunkeei *EABW06. Survival rates (%) at pH 3.0 and pH 6.5 (control) over 3 h. Values represent mean ± standard deviation (SD) of three independent experiments

The survival of *A. kunkeei* EABW06 under simulated gastric conditions (pH 3.0) was evaluated over 3 h, with survival rates normalized to the initial viable count at 0 h (Fig. 1). Parallel experiments at pH 6.5 served as a non-acidic control. *A. kunkeei* EABW06 exhibited robust acid tolerance, retaining 97.9 ± 5.2% viability at 1.5 h and 96.4 ± 4.2% at 3 h under pH 3.0, compared to 101.5 ± 1.6% and 101.5 ± 2.7% survival, respectively, at pH 6.5. Statistical analysis (paired *t*-test, *p* > 0.05) revealed no significant difference in survival between the two pH conditions applied at both time points, indicating that acidic stress did not impair viability relative to the control.

**Fig. 2:**
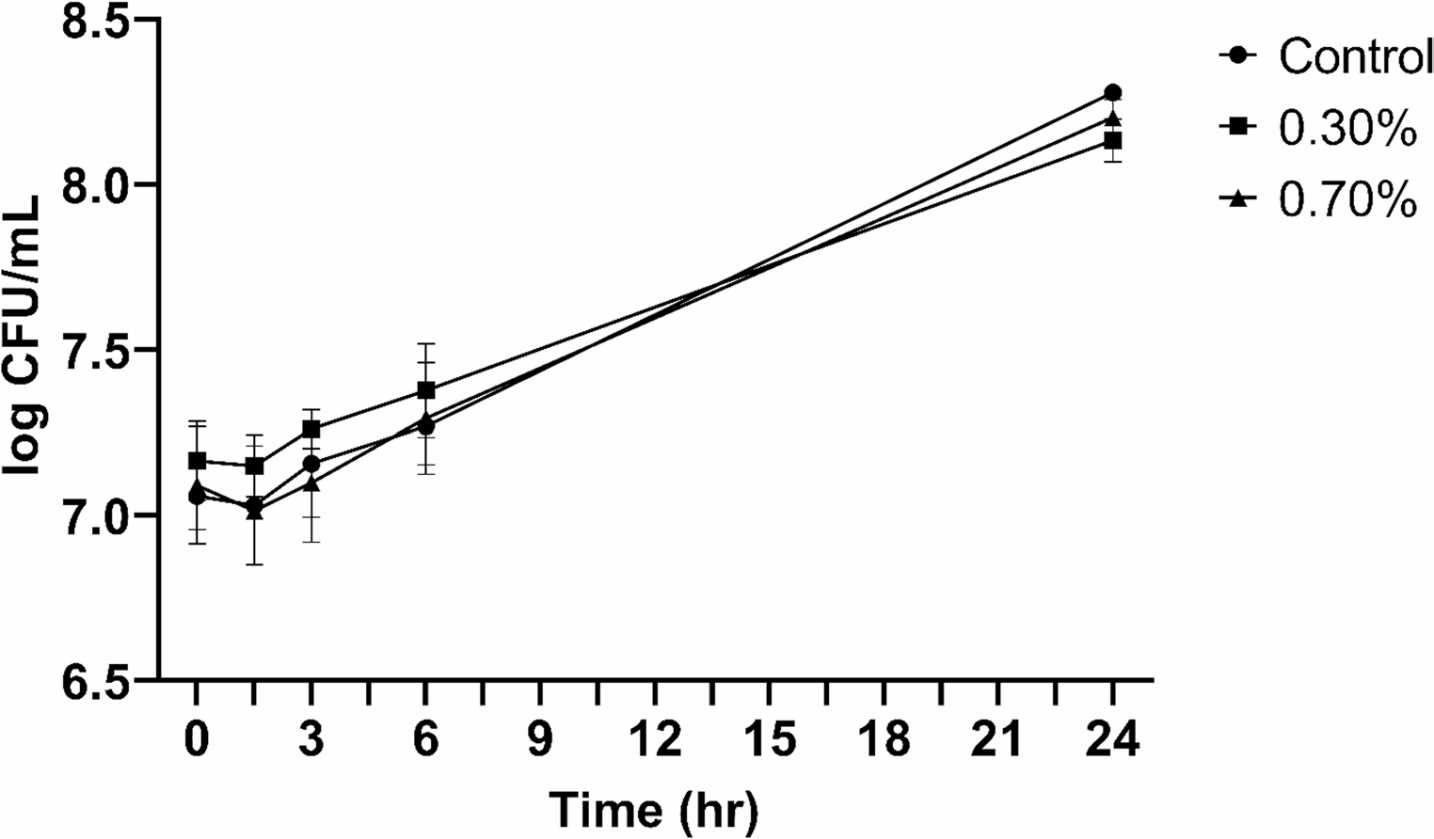
Growth of *A. kunkeei *EABW06 in Different Bile Salt Concentrations. The bacterial count (Log CFU/mL) was determined at different time points to assess bile salt resistance. Data represent the mean ± standard deviation (SD) of three independent experiments

To assess bile salt tolerance, viable cell counts (CFU/mL) were monitored over 24 h at 0.3% and 0.7% (w/v) bile concentrations (Fig. 2). Despite a slight reduction in viability compared to the bile-free control, no statistically significant differences (*p* > 0.05, one-way ANOVA) were observed between the control and bile-supplemented groups. After 24 h, viable counts reached 8.28 ± 0.02 log CFU/mL (control), 8.13 ± 0.06 log CFU/mL (0.3% bile), and 8.2 ± 0.05 log CFU/mL (0.7% bile). These results confirm that *A. kunkeei* EABW06 maintains metabolic resilience under bile stress, a critical trait for small intestinal colonization and probiotic functionality.

## Safety Profiling

### Hemolytic Activity

*A. kunkeei* EABW06 exhibited no hemolytic activity on blood agar plates, indicating the absence of β-hemolysis. This suggests that *A. kunkeei* EABW06 does not produce hemolysins, aligning with the safety criteria for potential probiotics. Similar findings were reported by Halder et al. [34], where Lactobacillus strains lacking hemolytic activity were considered safe for probiotic use.

### Antibiotic Susceptibility

The antibiotic susceptibility profile of *A. kunkeei* EABW06 was determined using the disc diffusion method. *A. kunkeei* EABW06 exhibited resistance to kanamycin, gentamicin, and vancomycin, while showing high sensitivity to chloramphenicol, clindamycin, and erythromycin (Table 3). Intermediate susceptibility was observed for tetracycline.

**Table 3:** Antibiotic susceptibility profile of *A. kunkeei* EABW06

Antibiotic	Zone of Inhibition (mm) ± SD	Interpretation*
Chloramphenicol (30 µg/disc)	29 ± 5.13	Susceptible (S)
Clindamycin (2 µg/disc)	33 ± 0.58	Susceptible (S)
Erythromycin (15 µg/disc)	29 ± 0.58	Susceptible (S)
Gentamicin (10 µg/disc)	8 ± 0.58	Resistant (R)
Kanamycin (30 µg/disc)	0 ± 0	Resistant (R)
Streptomycin (10 µg/disc)	9 ± 2.31	Resistant (R)
Tetracycline (30 µg/disc)	19 ± 1.15	Intermediate (I)
Vancomycin (30 µg/disc)	10 ± 0.58	Resistant (R)

* The interpretation of susceptibility (S), intermediate (I), and resistance (R) was based on the Clinical and Laboratory Standards Institute (CLSI) guidelines

### Cytotoxicity Assessment of *A. kunkeei *EABW06 CFS on Caco-2 Cells

**Fig. 3:**
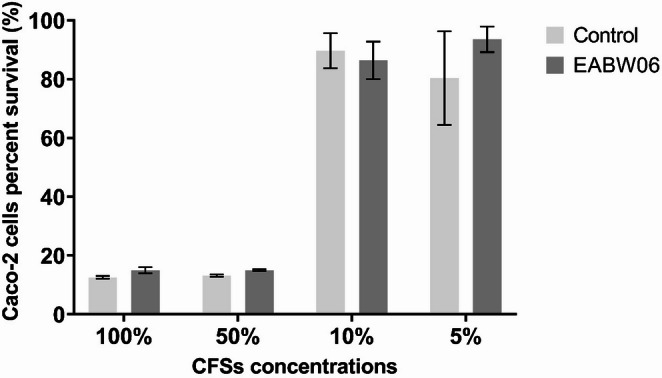
Cytotoxicity assay of *A. kunkeei* EABW06 CFS on Caco-2 cells. Caco-2 cell survival percentage relative to the control in the presence of bacterial culture cell-free supernatant (CFS) at varying concentrations (100%, 50%, 10%, and 5% v/v). The control used is sterile MRS broth at equivalent concentrations. Values are expressed as mean ± SD of three replicates

The survival rate of Caco-2 cells after CFS exposure is reported in Fig. 3. At all the concentrations tested, no significant differences (*p* > 0.05) in the survival rate between the control and the tested CFS were observed, confirming that the CFS from *A. kunkeei* EABW06 did not exhibit anti-proliferative effects on human intestinal epithelial cells, suggesting its non-cytotoxic nature.

## Technological Characteristics

### Acidification Profile

**Fig. 4:**
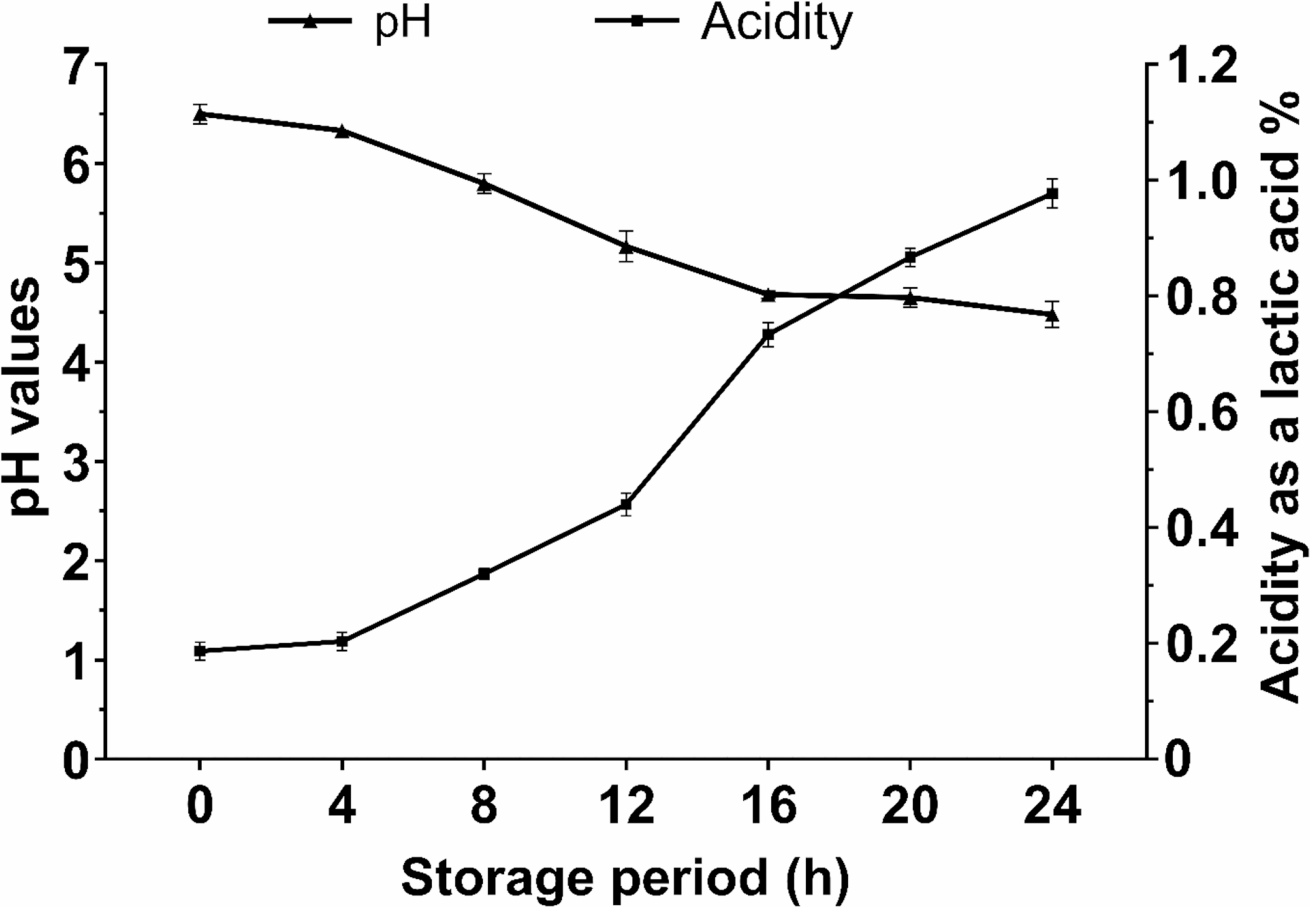
Acidification profile of skim milk fermented by *A. kunkeei *EABW06 during 24-hour incubation at 30 °C. The profile shows the changes in pH (left Y-axis) and titratable acidity (right Y-axis), expressed as % lactic acid, measured at 0, 4, 8, 12, 16, 20, and 24 h. Data represent the mean ± standard deviation (SD) of triplicate experiments

The acidification kinetics of *A. kunkeei* EABW06 in milk were monitored over 24 h (Fig. 4). The strain demonstrated active metabolism, progressively reducing the pH from an initial value of 6.50 ± 0.10 to a final pH of 4.48 ± 0.13. A notable decrease in pH to 4.68 ± 0.03 occurred within 16 h. Concurrently, titratable acidity increased significantly from 0.19 ± 0.02% to 0.98 ± 0.02% lactic acid over the fermentation period.

However, when evaluated against the pH-based criteria established by Ayad et al. [52], which classifies strains as fast (ΔpH ≥ 0.4 unit within 3 h), medium (3–5 h), or slow (> 5 h) acidifiers, *A. kunkeei* EABW06 demonstrates a slow acidification profile. The strain required > 12 h to achieve a ΔpH of 0.4 units from the initial pH. This indicates that while the strain eventually produces high acidity, its initial acidification rate in milk is comparatively slow, a characteristic that may be advantageous for its potential use as an adjunct culture rather than a primary starter.

### Antioxidant Activity of *A. kunkeei *EABW06

**Fig. 5:**
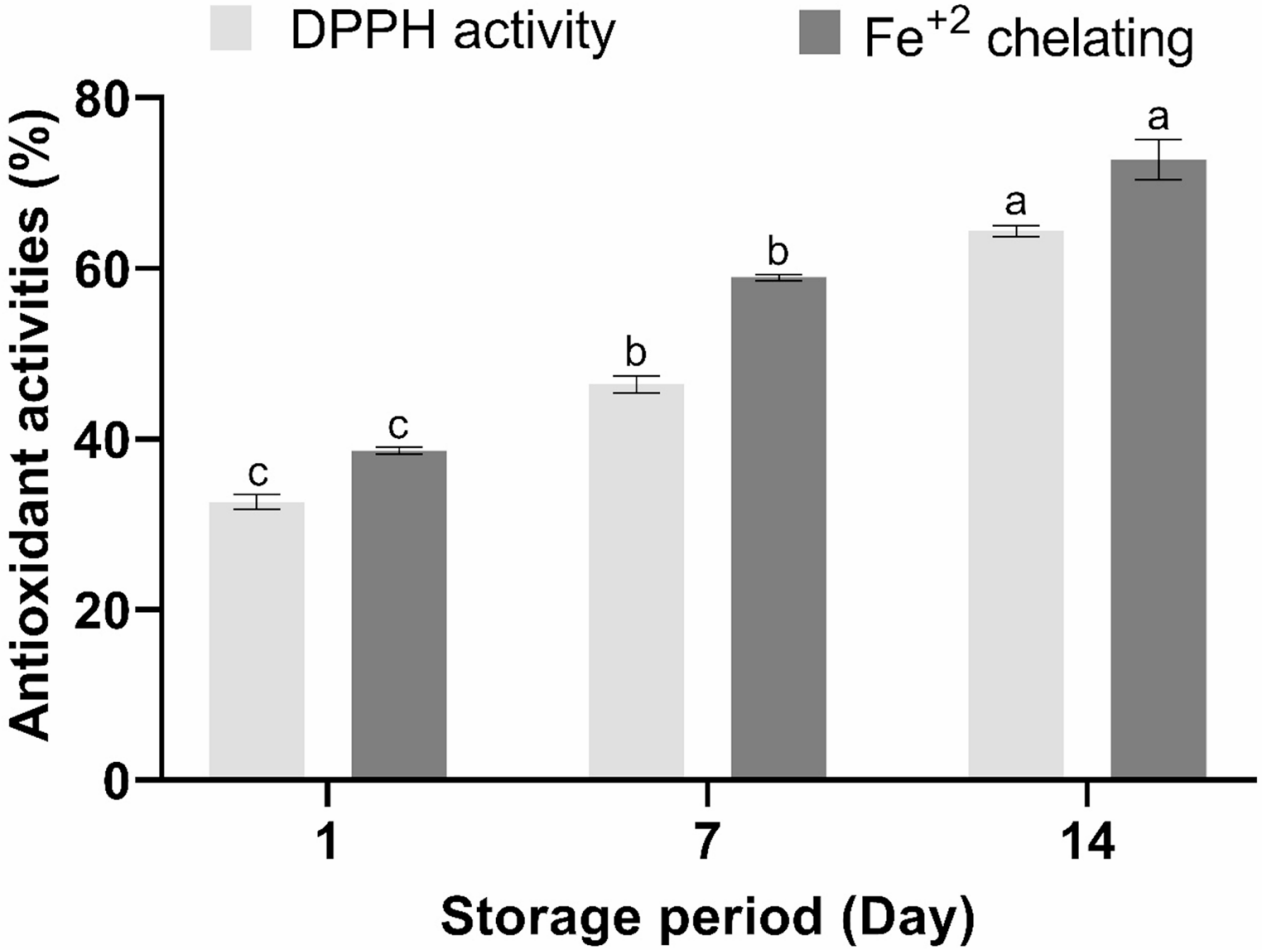
Antioxidant activity of *A. kunkeei *EABW06 in fermented milk during storage. The water-soluble extract (WSE) was assessed for DPPH radical scavenging activity and Fe²⁺ chelating ability. Data represent the mean ± standard deviation (SD) of triplicate experiments. Different lowercase letters (a, b, c) indicate statistically significant differences (*p* < 0.05) between storage days for each assay, as determined by one-way ANOVA followed by Tukey’s post-hoc test

The antioxidant capacity of WSE was determined using DPPH radical scavenging and Fe^+2^ chelating methods and the results are depicted in Fig. 5. *A. kunkeei* EABW06 exhibited antioxidant ability by two techniques either on day one or after 14 days of cold storage. The DPPH scavenging activity value mean values at day one was (32.67 ± 0.9%), while after 14 days of cold storage was (64.4 ± 0.6%) confirming that the antioxidant activity of the fermented milk is in the increasing trend with the increase of storage period. In addition, *A. kunkeei* EABW06 showed a good chelating ability of Fe^2+^ even at day one (38.63 ± 0.45%), or after 14 days of cold storage (72.73 ± 2.36%). Statistically, antioxidant ability of WSE was significantly (*p* < 0.05) affected by storage period.

### Proteolytic Activity

**Fig. 6:**
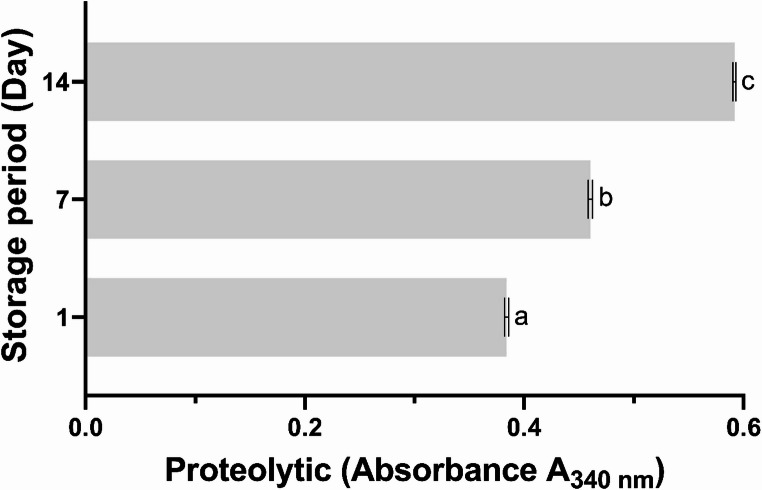
Spectrophotometric quantification of proteolytic activity by *A. kunkeei *EABW06. The release of free α-amino groups from casein hydrolysis in fermented milk was measured using the o-phthaldialdehyde (OPA) assay at 340 nm. Data represent the mean ± standard deviation (SD) of triplicate experiments. Different lowercase letters (a, b, c) indicate statistically significant differences (*p* < 0.05) between storage days, as determined by one-way ANOVA followed by Tukey’s post-hoc test

The proteolytic activity of *A. kunkeei* EABW06 was determined by quantifying the release of free α-amino groups in the fermented milk (Fig. 6). The strain demonstrated substantial proteolytic activity, with an absorbance value of 0.38 ± 0.002 at 340 nm on day 1, suggesting a potential role in flavor and aroma development. This activity increased significantly (*p* < 0.05) to 0.59 ± 0.002 after 14 days of cold storage, indicating a progressive breakdown of casein and release of peptides and amino acids over time.

### Organic Acids Produced by *A. kunkeei* EABW06

**Fig. 7:**
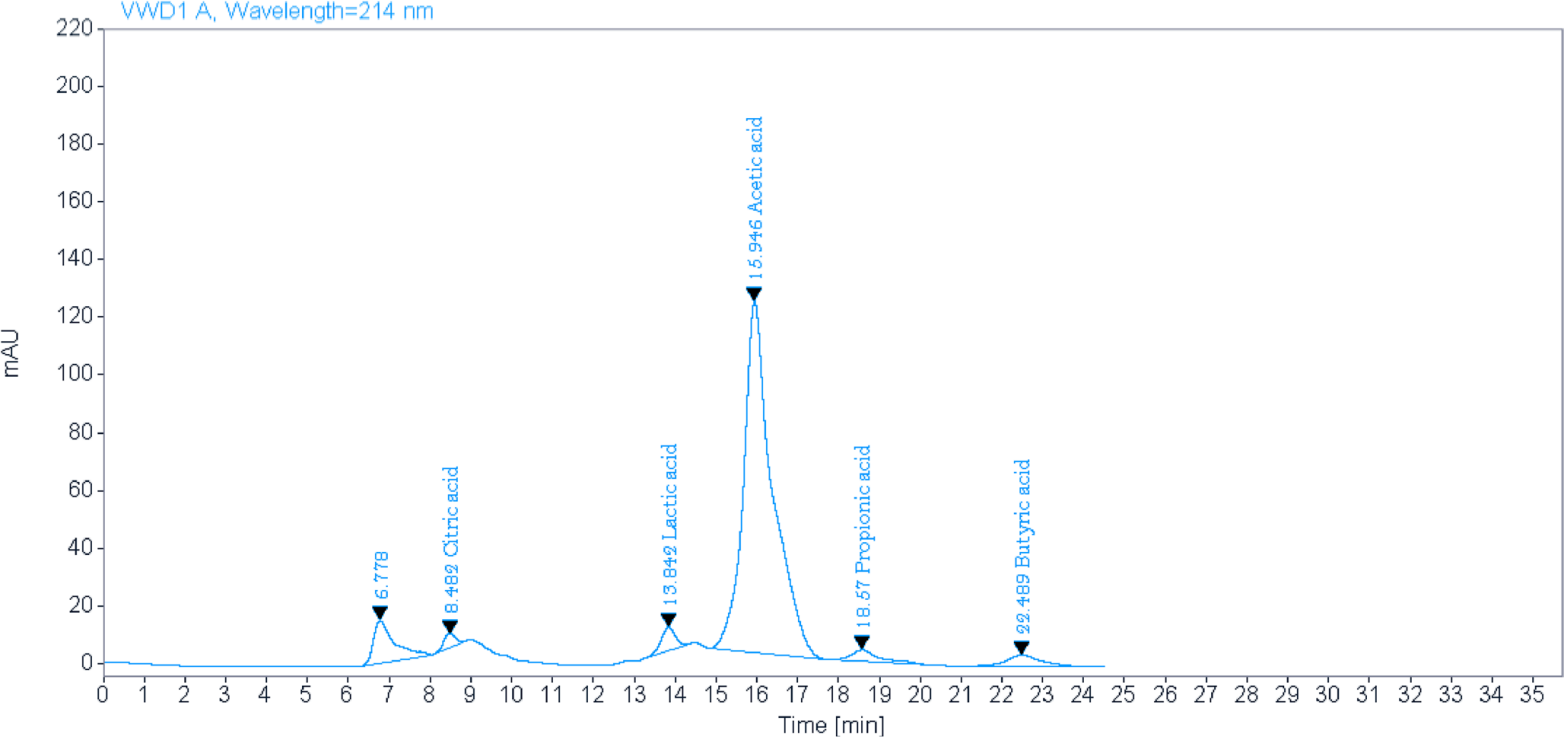
HPLC-RID chromatogram showing the organic acid profile of skim milk fermented by *A. kunkeei* EABW06

The organic acid metabolites produced by *A. kunkeei* EABW06 in the fermented milk were quantified using HPLC (Fig. 7). Acetic acid (4572.1 mg/L) and lactic acid (1457.2 mg/L) were the dominant metabolites, consistent with the homofermentative and fructophilic metabolism of the species. Notably, butyric acid was also detected at a considerable concentration (1461.1 mg/L), while propionic (1052.3 mg/L) and citric (128.5 mg/L) acids were present in lower amounts. This profile, characterized by high concentrations of antimicrobial organic acids, provides a mechanistic basis for the strain’s previously observed antimicrobial and antifungal activity.

## Discussion

Four isolates from hive-derived materials (pollen grains, beebread, and beeswax) were characterized by 16 S rRNA gene sequencing. Two pollen‐derived strains (EAPG01, EAPG02) and one beebread isolate (EABB05) were identified as of the *Lacticaseibacillus paracasei/casei* clade with ≥ 96% coverage and 100% identity, reflecting the known difficulty in resolving these closely related species by 16 S alone [53, 54]. The beeswax‐derived isolate (EABW06) was unambiguously identified as *Apilactobacillus kunkeei*, a fructophilic lactic acid bacterium adapted to high‐fructose niches such as honey and bee guts [55, 56]. Based on its unique ecological origin and clear taxonomic assignment, *A. kunkeei* EABW06 was selected for further probiotic and technological characterization. FLABs thrive in fructose-rich niches, a trait linked to their production of bioactive metabolites like phenyllactic acid, which exhibit antimicrobial and immunomodulatory properties [20, 21]. The co-isolation of *Lactobacillus paracasei* from pollen grains further underscores the ecological versatility of LAB in bee-associated ecosystems, supporting prior studies that identified lactobacilli in honeybee guts, pollen, and beeswax [57, 58]. These niches—characterized by high osmotic pressure, low pH, and antimicrobial compounds—likely drive LAB evolution toward stress tolerance and niche-specific functionality, positioning them as promising probiotics.

*A. kunkeei* EABW06 demonstrated broad-spectrum antimicrobial activity against *E. coli* O157:H7, *S. typhi*, and *C. difficile*, consistent with LAB-mediated pathogen inhibition via organic acids (lactic, acetic), hydrogen peroxide, and bacteriocin-like substances [59]. Organic acids are recognized as major antimicrobial effectors in lactic acid bacteria, as they suppress pathogens by lowering environmental pH. Bacteriocins, in contrast, act through membrane disruption and interference with microbial metabolic processes. Previous studies have reported that certain *A. kunkeei* strains produce kunkecin A, a nisin-like bacteriocin active against Gram-positive pathogens [60]. Additionally, Butler et al. [61] showed that *A. kunkeei* may secrete extracellular proteins under stress conditions, which could contribute to antimicrobial activity. Although bacteriocin production was not directly investigated in our study, it remains a plausible mechanism that warrants further exploration. In our work, the inhibition of *A. fumigatus* (23 mm), *A. flavus* (29 mm), and *C. albicans* (18.8 mm) could be attributed to phenyllactic acid, a well-known antifungal metabolite produced by several *A. kunkeei* strains, suggesting that organic acids are the most likely contributors to the observed antifungal activity. Future studies will be needed to confirm whether bacteriocin production plays a role in the antimicrobial profile of strain EABW06.

The potent antimicrobial and antifungal activity observed for *A. kunkeei* EABW06 finds a clear mechanistic explanation in its distinctive organic acid profile. Quantification via HPLC revealed that the strain predominantly produced acetic acid (4572.1 mg/L) and lactic acid (1457.2 mg/L) in fermented milk. The high yield of acetic acid is a hallmark of the fructophilic metabolism of *A. kunkeei*. This species efficiently converts fructose or other substrates into acetate instead of ethanol, a distinctive feature of its obligately heterofermentative pathway [21, 62]. Acetate is a compound known for its broad-spectrum antimicrobial potency, especially against fungi and Gram-negative bacteria [63, 64].

A particularly notable finding was the considerable production of butyric acid (1461.1 mg/L), a metabolite less commonly reported as a major product in LAB fermentations [65, 66]. Interestingly, Simsek et al. [67] isolated two FLAB strains — *Fructobacillus tropaeoli* R581701 and *Apilactobacillus kunkeei* TR481701 — that also produced butyric acid, alongside lactic and acetic acids under their culture conditions. In that work, *A. kunkeei* TR481701 showed an approximate acetate: butyrate ratio close to 3:1, a proportion similar to that observed in our study. Butyric acid is a potent antimicrobial agent, known to reduce bacterial colonization and suppress inflammation [68]. This multi-faceted acidic profile, complemented by propionic and citric acids, creates a synergistic antimicrobial environment [69].

*A. kunkeei* EABW06 exhibited exceptional gastrointestinal resilience, surviving > 96% at pH 3.0 and thriving in 0.3–0.7% bile salts. These results align with studies on FLAB adaptations to acidic and bile-rich environments [21, 26, 70]. The acid tolerance of *A. kunkeei* EABW06 surpasses that of the well-known probiotic *Lactobacillus rhamnosus* GG (80–90% survival under similar conditions), highlighting its potential as a next-generation probiotic [71]. Such robustness is critical for survival in the GIT and subsequent colonization—a prerequisite for probiotic efficacy. Additionally, recent studies have reinforced the probiotic potential of *A. kunkeei* by highlighting its strong antimicrobial and stress-tolerance properties. For example, Usta et al. [72] reported that *A. kunkeei* strains isolated from *Apis mellifera anatoliaca* and *Bombus terrestris* exhibited antimicrobial activity along with key probiotic traits such as pathogen inhibition, acid tolerance, and adhesion capacity. These findings are consistent with our results, further supporting the role of *A. kunkeei* as both a bee-associated symbiont and a promising candidate for probiotic applications.

The absence of hemolytic activity and cytotoxicity towards Caco-2 cells in *A. kunkeei* EABW06 represents an important indicator of its safety profile. These in vitro assays are widely recognized as fundamental steps in the strain specific safety assessment of candidate probiotics [73]. Our results confirmed that *A. kunkeei* EABW06 is non hemolytic and non-cytotoxic to Caco-2 cells. Such findings support its potential safety for probiotic applications, as preservation of host cell viability is a critical parameter in probiotic selection.

In terms of antibiotic susceptibility, the strain was resistant to kanamycin, gentamicin, and vancomycin, traits that are generally considered intrinsic and common within lactobacilli [74]. Importantly, the strain remained susceptible to several clinically relevant antibiotics, including chloramphenicol, clindamycin, and erythromycin. This profile aligns with the strain-specific resistance patterns documented in *A. kunkeei*. For instance, Vergalito et al. [63] reported that *A. kunkeei* strains K18 and K34 exhibited resistance to kanamycin and ampicillin. Similarly, Simsek et al. [67] observed an intrinsic resistance profile to aminoglycosides, including gentamicin, kanamycin, and streptomycin, in *A. kunkeei* strain TR481701. The resistance patterns observed in EABW06 may therefore represent intrinsic traits of *A. kunkeei* rather than evidence of transferable resistance genes. This distinction is crucial to ensure that probiotic applications do not contribute to the spread of antibiotic resistance. Overall, the absence of hemolytic activity together with the antibiotic susceptibility profile supports the potential use of *A. kunkeei* EABW06 as a safe probiotic candidate. Nevertheless, further genomic analyses are recommended to verify the absence of transferable antibiotic resistance genes, thereby strengthening the evidence for its safety in human consumption [75, 76].

Rapid acid production by LAB is one of the primary criterion in the selection of starter cultures used for food fermentation technology [77], as that is essential for lowering the pH and, thus, inhibiting the growth undesirable bacteria during the initial stage of fermentation. From the technological point of view, the acid production abilities of *A. kunkeei* EABW06 were very low as it took 16 h incubation to reduce the pH of milk to < 5.0. This slow acidification rate suggests that while *A. kunkeei* may not serve as a primary starter culture, it holds promise as an adjunct culture in dairy fermentations, contributing to flavor development and potentially enhancing the sensory attributes of fermented products. This aligns with findings by Molina et al. [78], who characterized *A. kunkeei* NFICC 2128 and highlighted its metabolic adaptability in utilizing various fermentable substrates, thereby supporting its application in diverse fermentation processes.

Previously, Taha et al. [79] suggested that the oxidative stability of fermented milk depends on the antioxidant peptides released during the fermentation of milk by LAB strains. These peptides act as electron donors and can react with free radicals to convert them to more stable products. Also, the protective ability of probiotics against oxidative stress has been elucidated by ROS scavenging, chelation of metal ion, and the reduction of ascorbate autoxidation as reported by Amaretti et al. [80]. The antioxidant components in LAB are bacterial exopolysaccharides, bioactive peptides, antioxidant enzymes, and manganese ions. Moreover, the gut microflora can produce bioactive dietary antioxidants through bioconversion processes using various enzymatic reactions. Such attributes not only improve the shelf-life and nutritional value of fermented foods but also offer potential health benefits by mitigating oxidative stress in consumers. Further, the increase in antioxidant potential during the refrigerated storage was probably caused by proteolysis process, which occurs in the fermented skimmed milk and increases the contents of organic acids as reported by Halah, Mehanna [81]. Consequently, the antioxidant capacities of *A. kunkeei* EABW06 suggested that they could be highly beneficial in the process of food fermentation.

Proteolysis is essential for the breakdown of milk proteins into peptides and amino acids, which contributes to the development of flavor and texture in fermented dairy products. Moreover, the peptides released during proteolysis may possess bioactive properties, including antioxidant activity, ACE-I activity, thereby enhancing the functional value of the fermented product [82]. *(A) kunkeei* EABW06 exhibited elevated proteolytic activity that intensified with cold storage, due to the presence of proteinases and peptidases, which hydrolyze proteins to large, small peptides and amino acids. The obtained results were similar with Li et al. [83] who suggested that the proteolytic performance of fermented milk produced by *S. thermophilus* in co-cultures with *(B) animalis* subsp. lactis or *L. plantarum* was modified during the 21 days of refrigerated storage furthermore, all of the fermented milks exhibited increasing proteolysis with extended time. Correlation of antioxidant and proteolytic activity was measured. *A. kunkeei* EABW06 showed a positive correlation coefficient (0.851) between proteolytic activity and radical scavenging activity. The difference of radical scavenging activities among hydrolysates may be attributed to the amino acid compositions, molecular distribution, their structures and sequences of peptide. A few studies [84, 85] related to the production of antioxidant peptides in milk fermented with LAB indicate that the development of radical scavenging activity is a strain-specific characteristic, and radical scavengers are related to proteolysis. Our result was similar to Li et al. [86] who reported that the antioxidant activity of CGM protein hydrolysates was related to the concentration and molecular weight of hydrolysates. In summary, *A. kunkeei* EABW06 exhibits desirable technological properties, including controlled acidification, antioxidant activity, and proteolytic capacity, making it a promising adjunct culture for dairy fermentations. These functional attributes not only enhance the sensory qualities of fermented products but also contribute to their potential health benefits.

## Conclusion

This study establishes *A. kunkeei* EABW06, isolated from beeswax, as a promising probiotic and biopreservative candidate. *(A) kunkeei* EABW06 demonstrated essential probiotic traits, including robust tolerance to acidic pH and bile salts. In addition, it exhibited significant antimicrobial and antifungal activities, in some cases even surpassing the activity of the reference drug Amphotericin (B) This activity is linked to a unique metabolic profile characterized by high acetate and notable butyrate production, creating a synergistic acid cocktail that can enhance the microbial safety and shelf-life of foods.

*A. kunkeei* EABW06 also showed antioxidant and proteolytic activities. These traits enhance its appeal for use in functional foods that combat oxidative stress. In dairy products, they may improve flavor and texture, suggesting that this strain could be a promising option for accelerating cheese ripening. Moreover, the absence of hemolytic activity and its safe antibiotic resistance profile support its suitability for human consumption.

Overall, our results provide a solid foundation for future in vivo studies to confirm safety and efficacy, as well as investigations into the genome and fermentation optimization. *A. kunkeei* EABW06 could thus be harnessed for applications in food preservation, health, and possibly therapeutic uses.

## Data Availability

All data supporting the findings of this study are available within the article.
